# Poorly cohesive adenocarcinoma of the ampulla of Vater: a case report

**DOI:** 10.1186/s40792-016-0142-9

**Published:** 2016-02-15

**Authors:** Hayato Yamauchi, Shinji Sakurai, Kei Hagiwara, Tomonori Yoshida, Yuichi Tabe, Takaharu Fukasawa, Shinsuke Kiriyama, Minoru Fukuchi, Hiroshi Naitoh, Hiroyuki Kuwano

**Affiliations:** Department of Surgery, Japan Community Health Care Organization, Gunma Chuo Hospital, 1-7-13 Kouun-cho, Maebashi, Gunma 371-0025 Japan; Department of Diagnostic Pathology, Japan Community Health Care Organization, Gunma Chuo Hospital, Gunma, Japan; Department of General Surgical Science (Surgery I), Gunma University, Graduate School of Medicine, Gunma, Japan

**Keywords:** Ampullary cancer, Cohesive adenocarcinoma, Poorly differentiated adenocarcinoma

## Abstract

A 47-year-old Japanese male was submitted to pancreaticoduodenectomy for an ampullary cancer. Pathologically, the ampullary cancer was poorly cohesive adenocarcinoma without tubular structure. Moreover, locoregional lymph nodes were swollen with hypervascularity, plasmacytes infiltration, and hemorrhage. Our case seems to be different from usual poorly differentiated adenocarcinoma.

## Background

Ampullary carcinoma is a malignant tumor arising in the last centimeter of the common bile duct, and patients with these tumors have been reported to have a relatively favorable prognosis after surgical resection [1]. Among the tumors originated from the ampulla of Vater, poorly differentiated adenocarcinoma is a very rare disease [2] and the clinical outcomes of the patients are undetermined. On the other hand, Castleman’s disease (CD) is a lymphoproliferative disorder which was first described by Dr. Benjamin Castleman in 1956 and which often develops in the retroperitoneal lymph nodes [3].

Here, we present a rare case of poorly cohesive adenocarcinoma of the ampulla of Vater without tubular structure, the histology of which is similar to poorly cohesive adenocarcinoma of the stomach. The swollen locoregional lymph nodes showed marked plasma cells infiltration, vascular proliferation like CD and hemorrhage, but no lymph node metastasis of the tumor was found.

## Case presentation

The patient was a 47-year-old man who had no pathological antecedents. He admitted to our hospital with hyperbilirubinemia of 1.6 mg/dl at health screening, with appetite loss and epigastralgia appearing 6 months ago. Carbohydrate antigen 19-9 and carcinoembryonic antigen were normal. Abdominal computed tomography and magnetic resonance imaging showed a dilatation of the common bile duct and a stone in the gallbladder. Neither tumor nor lymph nodes swelling were observed. No stones or abnormal arrangement of the pancreaticobiliary ductal union were found by an endoscopic retrograde cholangiopancreatography. Gastrointestinal endoscopy showed irregularly shaped concavity on the ampulla of Vater, and histology of the biopsy revealed signet ring cell carcinoma.

**Fig. 1:**
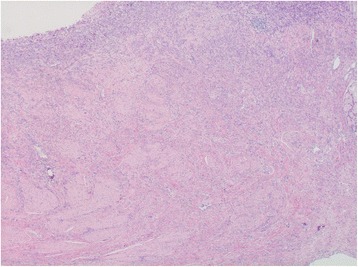
Infiltrating carcinoma cells shows scirrhous appearance in the ampulla of Vater

**Fig. 2:**
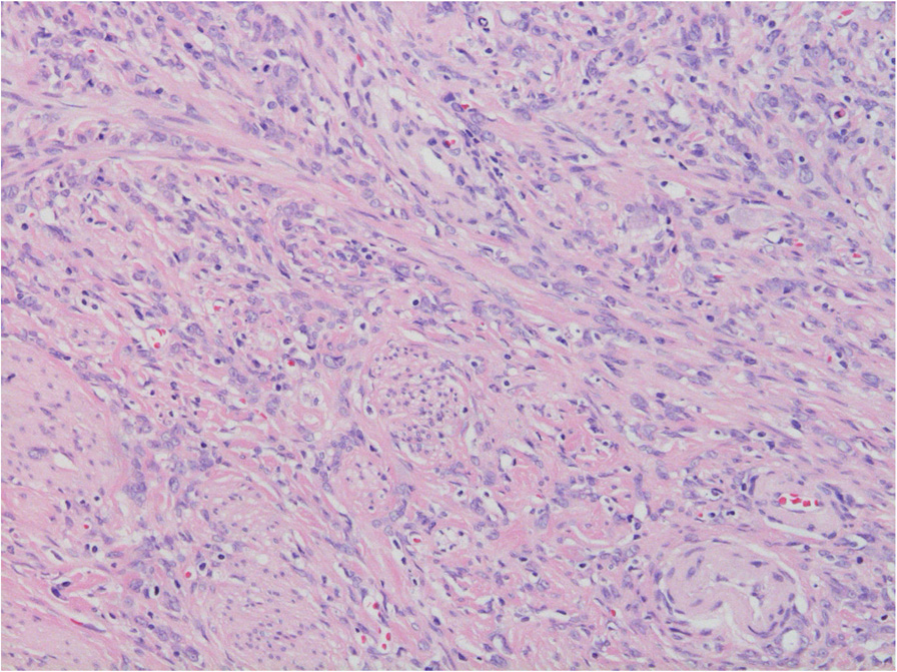
The proliferating tumor cells consist of poorly cohesive adenocarcinoma without tubular structure

A pancreatoduodenectomy was performed. The final pathological diagnosis was poorly cohesive adenocarcinoma including signet ring cell carcinoma component in the Vater’s ampulla without lymph node metastases (Fig. 1). The carcinoma consisted of complete poorly differentiated carcinoma cells most of which showed non-cohesive pattern, and tubular structure was not seen in any sections examined for pathological analysis (Fig. 2). Tumor invasion was seen around the common duct, bile duct, and pancreatic duct in the ampulla of Vater and the duodenum wall. A little lymphatic and vascular invasion was observed. Perineural invasion was little seen.

**Fig. 3:**
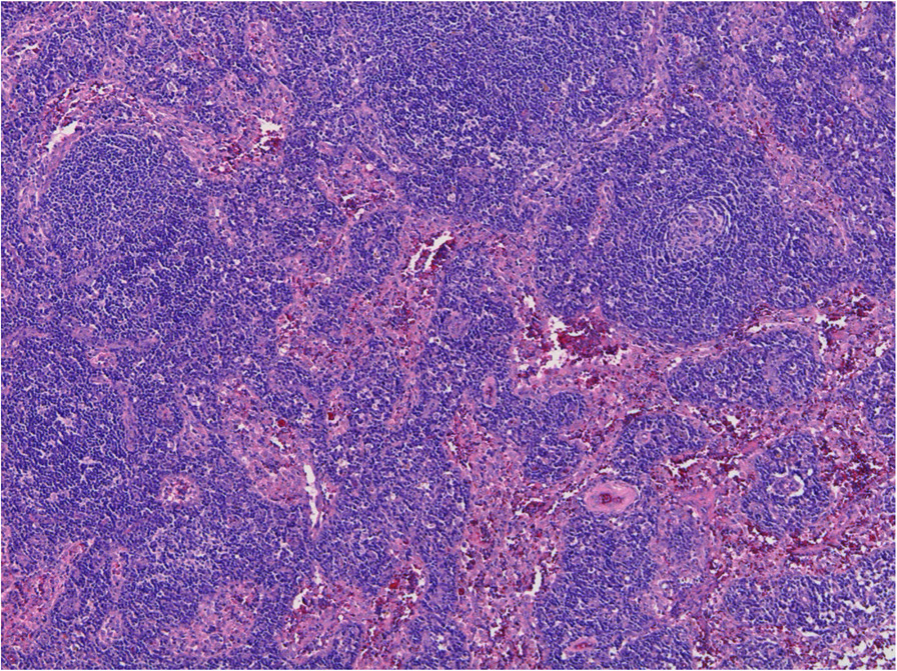
Lymph follicles were atrophic, and vascular hyperplasia was found in the germinal centers

**Fig. 4:**
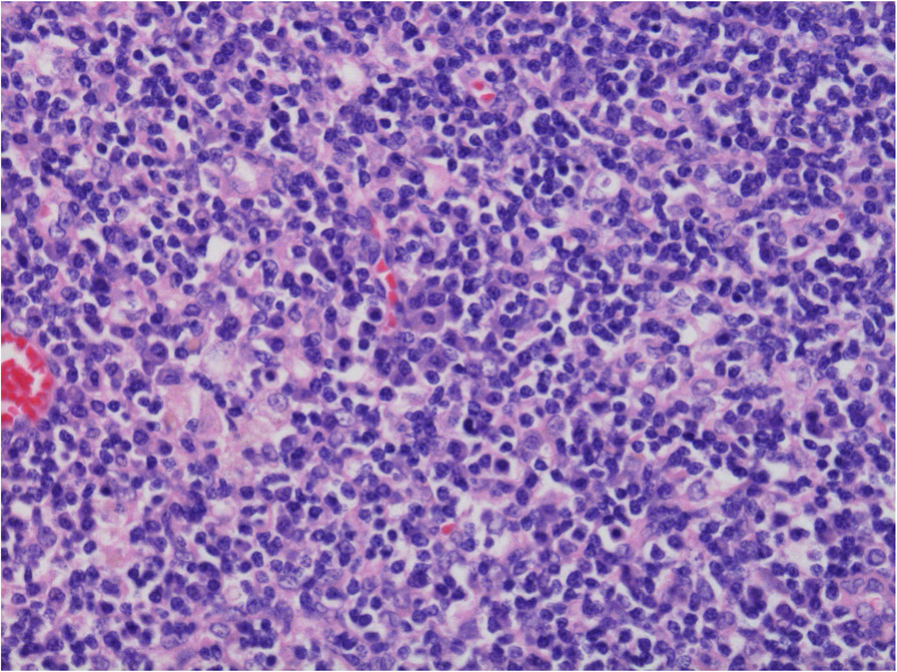
Marked plasma cell infiltration was observed in the interfollicular stroma

The locoregional lymph nodes were swollen with hemorrhage in the capsule. Lymph follicles were atrophic, and vascular hyperplasia was found in the germinal centers (Fig. 3). Marked plasma cell infiltration was seen in the interfollicular stroma (Fig. 4). However, hyalinization of the vascular wall was not seen. Histological findings resemble those of CD but are slightly different. Immunohistochemically, plasma cells in the interfollicular region were evenly positive for kappa and lambda light chain, which meant no evidence of clonal proliferation.

According to the TNM classification, the tumor was classified as pT3 N0 M0 ly1 v1 pn0 pstageIII. The patient remained well and had no evidence of locoregional and metastatic recurrence during the 28 months of follow-up.

### Discussion

The primary adenocarcinoma of the ampulla of Vater is a rare tumor, and most ampullary adenocarcinomas are well differentiated [2]. Ampullary carcinomas are thought to arise from the glandular epithelium of the ampulla of Vater [4]. It has been suggested that molecular biologically ampullary carcinoma is different from bile duct and pancreatic carcinomas, whereas that share the same molecular biological characteristics with duodenal carcinomas [1]. The clinical outcome of ampullary carcinoma has been reported to be better than those of bile duct and pancreatic carcinomas [1]. Thus, it would be important to distinguish the origin of carcinomas developed around the region of the ampulla of Vater.

The present case was diagnosed as ampullary carcinoma, since the tumor invaded both the common bile duct and the biliary and pancreatic ducts in the duodenal wall. However, the tumor in our case was non-cohesive type of poorly differentiated adenocarcinoma, in which glandular structure was not observed in any of specimens, and the histology resembled scirrhous carcinoma of the stomach, which was classified into poorly cohesive carcinoma according to World Health Organization (WHO) classification. To our knowledge, this type of carcinoma is uncommon in any of the ampulla of Vater, pancreatic duct, and bile duct [2, 5]. Thus, poorly cohesive carcinoma was listed only in the stomach, but not in the bile duct, pancreas, and the ampullary region in WHO classification.

On the other hand, tumors of ampulla of Vater are categorized into bile duct carcinoma in Japanese classification [6]. Most extrahepatic cholangiocarcinomas have been reported to be histologically well-to-moderately differentiated adenocarcinomas [7]. As far as we researched in the PubMed, there has been no report about exclusively poorly cohesive adenocarcinoma of periampullary region. Thus, our case which consists of pure poorly cohesive carcinoma cells without tubular structure is thought to be quite rare.

However, our case was classified into poorly differentiated type of tubular adenocarcinoma (tub3) according to the General Rules for Surgical and Pathological Studies on Cancer of the Biliary Tract, Japanese Society of Biliary Surgery [6], because Japanese classification also did not have poorly cohesive carcinoma in the bile duct carcinoma as same as WHO classification.

Our patient underwent curative pancreatoduodenectomy. The poorly differentiated adenocarcinoma has been reported to exhibit higher probability of recurrence and show poor prognosis compared with other histological types after the curative resection [8–11]. The prognostic factors of ampullary carcinoma after resection include lymph node metastasis, pancreatic invasion, and perineural invasion [12, 13]. These histological findings are also the most important prognostic factors for bile duct carcinoma after curative resection [14, 15]. However, our case showed a little vascular invasion and perineural invasion without nodal metastasis in spite of the case of poorly differentiated carcinoma, which might indicate better prognosis than usual poorly differentiated adenocarcinoma.

Histopathologically, the cause of lymph node swelling was not due to the tumor metastasis but the CD-like change and hemorrhage. CD is a rare atypical lymphoproliferative disorder. And soon after the original presentation of Castleman et al., CD has been subdivided into a hyaline vascular and plasma cell histopathological pattern, with intermediate variants [16]. The pathological findings of the locoregional lymph nodes in our case revealed hypervascular germinal centers with marked interfollicular plasma cell infiltration, which are common features of CD. However, hyalinization of the proliferating vessel was not observed, which was different from CD. The patient has no past history related to immune dysregulation. Therefore, we could not definitely diagnose the patient as CD.

## Conclusions

We report a rare case of poorly cohesive adenocarcinoma in the ampulla of Vater with enlarged lymph nodes like CD. The relation between clinicopathological features and the long-term outcomes are not clear only in this case. The histopathological findings of our case seem to be different from those of poorly differentiated adenocarcinoma, and it would be better to add poorly cohesive adenocarcinoma to the histopathological classification of the ampulla of Vater and bile duct.

## Consent

Written informed consent was obtained from the patient for publication of this case report and any accompanying images. A copy of the written consent is available for review by the Editor-in-Chief of this journal.

## Abbreviations

CD: Castleman’s disease
WHO: World Health Organization
